# The Impact of Homocysteine, Vitamin B12, and Vitamin D Levels on Functional Outcome after First-Ever Ischaemic Stroke

**DOI:** 10.1155/2017/5489057

**Published:** 2017-03-23

**Authors:** Merdin Markišić, Aleksandra M. Pavlović, Dragan M. Pavlović

**Affiliations:** ^1^General Hospital, Unit of Neurology, Svetog Save Street No. 33, Berane, Montenegro; ^2^Faculty of Medicine, University of Belgrade, Neurology Clinic, Clinical Center of Serbia, Dr. Subotica Street No. 6, 11 000 Belgrade, Serbia; ^3^Faculty for Special Education and Rehabilitation, University of Belgrade, Visokog Stevana Street No. 2, 11 000 Belgrade, Serbia

## Abstract

We explored the relationship between acute ischaemic stroke (IS) early functional outcome and serum levels of homocysteine, vitamin B12, and D in a noninterventional prospective clinical study. We enrolled 50 patients with first-ever IS and performed laboratory tests and functional assessment at three time points: on admission and three and six months after stroke. Modified Rankin Scale (mRS), NIHSS scale, and Barthel index (BI) scores were assessed in all participants by trained examiner blinded to laboratory data. Patients did not receive treatment that might alter laboratory data. Admission NIHSS correlated with homocysteine levels (*r* = 0.304, *p* < 0.05), B12 level (*r* = −0.410, *p* < 0.01), and vitamin D levels (*r* = −0.465, *p* < 0.01). Functional outcome measures (BI and mRS) did not significantly correlate with homocysteine and vitamin D3 levels at 3 and 6 months. However, a positive correlation with vitamin B12 levels was detected for BI both at 3 and 6 months and mRS at 6 months. Higher serum vitamin B12 levels were associated with better functional outcome at follow-up.

## 1. Introduction

Stroke remains the third leading cause of death after heart diseases and malignancies and the most frequent cause of disability in people after 60 years of age worldwide, in spite of extensive research and introduction of preventive measures [1, 2]. Ischaemic stroke (IS) is mostly preventable and risk factors are similar both in developed and in undeveloped countries [3]. Treatable conditions comprise around 90% of all risk factors, including arterial hypertension, diabetes mellitus, cardiac diseases, cigarette smoking, obesity, hyperlipidemia, physical inactivity, alcohol overuse, unhealthy diet, psychosocial stress, and depression [3, 4]. Although extensive, this list is not complete and new potentially treatable factors contributing to stroke risk are emerging continuously: hyperhomocysteinemia, vitamin B12 and vitamin D deficiencies, thyroid dysfunctions, and others [5]. There is also evidence that adequate management of vascular risk factors may be beneficial for functional outcome after stroke [5, 6].

Hyperhomocysteinemia increases the likelihood of stroke and is mostly dependent on folic acid (vitamin B9), vitamin B12, and vitamin B6 serum levels [7, 8]. Intestinal absorption of many food constituents decreases with aging and is probably one of the factors influencing vitamin deficiencies and hyperhomocysteinemia, although genetic polymorphisms may also contribute to metabolic disturbances [9]. It has been shown that homocysteine levels above 10.2 micromol/l (mcmol/l) double the risk of stroke [10], while with homocysteine levels above 20 mcmol/l the risk is fourfold [11]. Supplementation with vitamins B9, B12, and B6 appears to decrease the homocysteine levels and potentially contributes to stroke prevention although direct evidence is lacking [12, 13].

Vitamin B12 deficiency can be detected in 10–40% of the general population and may contribute to stroke and cognitive decline [14, 15]. Serum B12 levels may not be sufficient measures of B12 status but should be supported with homocysteine and methyl malonic serum levels [15]. Also, cut-off scores of vitamin B12 blood levels can be misleading as there is a wide discrepancy among experts setting the normal values in the range between 100 pmol/l and 350 pmol/l [16]. Vitamin B12 deficiency can be disclosed via elevated serum homocysteine levels at first and only later with decreased serum levels of vitamin B12; homocysteine levels should be kept in the safe range with continuous supplementation, bearing in mind that even B12 levels within the normal range can be associated with an increased risk of stroke twofold [14].

Vitamin D deficiency has been associated with a proinflammatory state that induces blood vessels injury but is also in relation to other vascular risk factors such as obesity, insulin resistance, autoimmunity, dyslipidemia, and oxidative stress [17–19]. Vitamin D deficiency has been related to an increased risk for stroke [18, 20]. In a cohort study from Hong Kong, in participants 45 years of age or older, a low vitamin D level was associated with increased risk of IS. The lowest risk was found at 25(OH)D levels between 70 and 80 nmol/l [21], while the optimal blood level is estimated to range from 75 to 100 nmol/l (30 to 40 ng/ml) [22]. Vitamin D status is influenced by at least another vascular risk factor, smoking, showing that the interplay of various risk factors is an important issue that should be addressed in stroke patients [23].

Previous studies investigated mainly vitamins role in secondary stroke prevention rarely focusing on potential impact on functional outcome after stroke, also eligible for an early intervention [24]. Hyperhomocysteinemia higher than 10 mcmol/l has been reported to be a risk factor for early poststroke neurological deterioration after 7 days, probably through neurotoxicity and epithelium injury in the vessel wall [25]. Low vitamin D status has also been suggested to be a risk factor for worse stroke prognosis and poor functional outcome [26]. Our study was designed to investigate the relationship between IS and serum status of homocysteine, vitamin B12, and vitamin D and go further to assess the importance of these correctable factors during the functional recovery three and six months after stroke, the time of the most evident improvement in stroke survivors.

## 2. Methods

### 2.1. Participants

Our study was a noninterventional prospective clinical study with 50 consecutive patients with acute IS, admitted to regional hospital in Berane, Montenegro. None of the patients were treated with rtTPA since it is not available in our hospital. Patients were assessed at the baseline and reassessed three and six months after stroke. Inclusion criteria comprised first-ever IS, age between 18 and 90 years, no previous injury to the central nervous system, and any other acute or chronic incapacitating medical disease. Only patients who survived the whole study period were included in the final analysis. Scales assessing neurological deficit and functional status and blood analyses were administered on admission and after three and six months after acute IS. Exclusion criteria for the study comprised previously known vitamin D and B12 deficiency and/or hyperhomocysteinemia or vitamin supplement therapy for other indications. Patients were treated for stroke risk factors and secondary stroke prevention as per current guidelines [27, 28]. Local Ethic Committee approved the study.

### 2.2. Neuroimaging

Computerized tomography of the brain was used to exclude hemorrhagic stroke and confirm diagnosis of IS.

### 2.3. Functional Measures

In our study, standard functional measures were used:

- Modified Rankin Scale (mRS) [29, 30]
- NIHSS scale [31, 32]
- Barthel index (BI) [33].

Evaluation was performed by trained examiner who was blinded to laboratory test results.

### 2.4. Blood Analysis

On all three visits morning fasting blood was drawn for determination of homocysteine, vitamin B12, and vitamin D levels.

### 2.5. Statistics

Along with descriptive statistics/measures, data was analyzed with independent samples *t*-test, Friedman test, and Wilcoxon test (for post hoc comparison), as well as Pearson correlation, using Statistical Package for the Social Sciences (SPSS) for Windows Version 16.0.

Follow-up 3- and 6-month analyses with mRS and BI as outcomes were adjusted for age and initial NIHSS.

## 3. Results

In our study group 22 (44%) participants were male and 28 (56%) female. Mean age was 71.96 ± 11.39, ranging between 45 and 90 years. Mean age was 72.79 ± 9.92 for females and 70.86 ± 13.28 for males, with no significant differences between sexes (*p* > 0.05). Clinical presentation comprised 25 (50%) cases with left-sided hemiparesis, 12 (24%) with right-sided hemiparesis, 10 (20%) with left-sided hemiplegia, and 3 (6%) with right-sided hemiplegia. Scores on NIHSS, BI and mRS are shown in Table 1. Admission NIHSS ranged from 5 to 22, mean 14 ± 7. According to the score on NIHSS, patients neurological deficit significantly improved over time of follow-up, which was also reflected in BI and mRS scores, that remained stable after 3-month follow-up visit.

Regarding laboratory data, homocysteine levels decreased and vitamin B12 and D levels increased during the follow-up (Table 2). Mean values of all parameters were within the pathological range.

Admission NIHSS correlated with homocysteine levels (*r* = 0.304, *p* < 0.05), B12 level (*r* = −0.410, *p* < 0.01), and vitamin D levels (*r* = −0.465, *p* < 0.01).

After adjustment for age and baseline NIHSS, serum homocysteine levels did not correlate with BI and mRS score at 3- and 6-month follow-up visit. However, correlation with BI at 6 months was at the level of statistical trend (*p* = 0.064) (Table 3).

Vitamin B12 levels significantly positively correlated with BI at 3 and 6 months and negatively with mRS levels at 6 months, after adjustment for age and NIHSS level. However, correlation with mRS at 3 months was at the level of statistical trend (*p* = 0.064) (Table 3).

No correlation was detected between vitamin D levels and BI and mRS at both time points, after adjustment for age and baseline stroke severity (Table 3).

## 4. Discussion

Results of study indicate significant association between serum homocysteine, vitamin B12, and vitamin D status and IS baseline severity. Also, a significant association was detected between vitamin B12 levels and parameters of early functional outcome after stroke. Since our patients did not receive any intervention except for standard secondary stroke prevention measures according to the current guidelines, we cannot conclude that vitamin supplementation and homocysteine reduction were associated with alteration of stroke outcome [27, 28]. However, early intervention may be of most importance as functional improvement after IS was noted to be most prominent in the first six months [34].

Homocysteine levels were decreasing in our IS patients during the period of observation compared to baseline levels. Our patients were not treated during the period of the study with vitamin supplementation that has been shown to reduce homocysteinemia and whether this is the result of diet changes is difficult to assess [13, 35]. Study of Wu and coworkers (2014) [36] showed that hyperhomocysteinemia in the first 72 hours after IS has negative prognostic impact on functional outcome after 6, 12, and 18 months, according to the scores on NIHSS, mRS, and BI. In this study elevated homocysteine levels were defined as >15 *μ*mol/l [36]. In our study hyperhomocysteinemia only correlated with worse functional outcome measured with BI at 6 months after stroke but at the level of statistical trend. Results of the Cilostazol in Acute Ischaemic Stroke Treatment (CAIST) study found homocysteinemia to be significant risk for early worsening of neurological status in first seven days after stroke [25]. In study by Mizrahi et al. (2005), no correlation between homocysteine level and functional outcome was detected when the Functional Independence Measure (FIM) tool was used [37], which could reflect the problem of different study designs and methodology for functional status assessment between different studies. Data on longer poststroke follow-up period are available from the study by Lee and coauthors showed that 18 months after IS patients with high homocysteine levels had significantly lower BI and higher NIHSS and mRS scores [35]. Interestingly, higher homocysteine group also had higher rate of reinfarction than the lower homocysteine group [36]. The fatigue scale score correlated with higher homocysteine serum level and lower BI as well [38].

In the study of Youssef and coworkers [39] homocysteine and C-reactive protein (CRP) levels significantly correlated with prognosis of cerebrovascular disease according to NIHSS and mRS scores. Similar results were obtained in the study of Tu and coauthors, who found that a high-sensitivity CRP and homocysteine were independent predictors of short-term outcome and mortality after IS, adjusted for age and NIHSS score [40]. In a retrospective study, Men and coworkers [41] reported an independent predictive value of elevated homocysteine and CRP after IS with unfavorable outcome after a month. Our analysis adjusted for age and baseline stroke severity showed that 6 months after a stroke BI scores negatively correlated with homocysteine levels at the level of statistical trend, with higher BI scores associated with lower homocysteine levels.

In the substudy of the Heart Outcomes Prevention Evaluation 2 (HOPE 2) study, a significant reduction of homocysteine levels due to supplementation with 2.5 mg of folic acid, 50 mg of vitamin B6, and 1 mg of vitamin B12 every day during five years led to significant lowering of stroke incidence in patients with cardiovascular risk factors [42]. Although reduced risk of nonfatal stroke was noted, homocysteine lowering did not influence the outcome 7 days after stroke according to the mRS score [42]. A meta-analysis published in 2010 confirmed effects of vitamin B supplementation on lowering the serum homocysteine levels but did not demonstrate a major effect in averting stroke [35, 43].

Most of the studies indicated that the risk of stroke diminishes significantly only with homocysteine levels lower than 10 mcmol/L [44–46]. Also, all-cause mortality from elevated homocysteine significantly decreased only when levels are less than 9 mcmol/L [46]. According to other data, atherosclerotic risk was elevated if serum homocysteine levels were ≥6.3 *μ*mol/L [47]. Additional data are available from the study by Hodis and coworkers, reporting that longstanding supplementation with high doses of B vitamins slows the progression of subclinical carotid atherosclerosis in people with homocysteine levels above 9 mcmol/L [48].

Intriguing finding is that vitamin B12 and D serum levels increased during the period of follow-up in our cohort, and no supplementation was offered to the patients. This could be secondary to dietary changes but our study was not designed to assess this.

An important correlation was found between vitamin B12 levels and functional status at all time points after stroke. A discrepancy between extracellular markers and intracellular biochemical reactions of vitamin B12 has been noted, with serum levels being significantly higher than cellular levels [49]. Consequently, apparently normal vitamin B12 serum level can be found in patients with vitamin B12 deficiency. The earliest marker of deficiency is low holotranscobalamin level followed by dysfunction of intracellular enzymes, followed by elevation of methylmalonic acid and homocysteine levels [49, 50]. In our group higher vitamin B12 levels correlated significantly with higher BI and lower mRS indicating better functional status, and the relationship remained significant 3 and 6 months after stroke. These findings disclose importance of vitamin B12 screening in stroke population.

There is some evidence that vitamin D deficiency is associated with an increased risk for vascular diseases and it also increases general mortality [19, 51]. In the study by Park and coworkers [19] only minority (13.6%) of patients had optimal vitamin D levels of ≥75 nmol/l at the time of stroke. A recent French study [52] also showed that vitamin D deficiency is very frequent in IS patients. On admission, our patients exhibited statistically significant correlation between vitamin D and NIHSS scores, indicating less deficit in patients with higher vitamin D serum levels. Supporting this is a finding from study by Turetsky and authors who detected by neuroimaging study of infarct volume that low vitamin D levels at the time of admission correlated with more tissue loss [53].

Mean baseline vitamin D levels remained decreased in our IS cohort. We failed to detect any correlation between vitamin D levels and functional outcome after stroke when analysis was adjusted for age and baseline NIHSS. Some researchers reported better outcome measured with BI in patients with higher vitamin D serum levels after three months [19]. Interestingly, an increased mortality one year after stroke in patients with low vitamin D levels on admission was registered, particularly in patients younger than 75 years of age [53].

There are some limitations of our study. Study group comprised small number of participants and therefore results should be interpreted with caution. This is a longitudinal registry of closely followed up patients with no missing data but with no control group involved. Since only surviving patients were included in the final analysis, we did not account for the competing mortality. We performed adjustment for age and baseline NIHSS data, but the *p* values were not adjusted for multiple testing.

## 5. Conclusion

Our findings indicate that serum homocysteine, vitamin B12, and vitamin D levels are associated with baseline first-ever stroke severity but also contribute to some extent to IS prognosis in early period after stroke. There is some evidence that early detection and management of these laboratory parameters may contribute both to primary and to secondary stroke prevention but whether interventions aimed at reducing homocysteine levels and increasing vitamin B12 and D levels would improve IS outcome remains to be elucidated in larger multicenter intervention studies.

## Figures and Tables

**Table 1:** NIHSS and functional scales (BI, mRS) at all time-points. Post hoc: Wilcoxon sign-rank test (Bonferroni correction).

	Admission	At 3 months	At 6 months	*p* value	
NIHSS	14 ± 7	12 ± 7	11 ± 7	<0.001	A, B, C
BI	72 ± 15	74 ± 16	75 ± 20	<0.001	A, B, C
mRS	4 ± 1	3 ± 1	3 ± 1	<0.001	A, B

NIHSS: National Institutes of Health Stroke Scale, BI: Barthel index, and mRS: Modified Rankin Scale.

А: admission versus at 3 months, B: admission versus at 6 months, and C: at 3 months versus at 6 months.

**Table 2:** Serum homocysteine, vitamin B12, and vitamin D levels at all time-points. Post hoc: Wilcoxon sign-rank test (Bonferroni correction).

	Admission	At 3 months	At 6 months	*p* value	
Homocysteine	12.6 ± 3.7	11.6 ± 3.0	10.9 ± 3.0	<0,001	A, B, C
Vitamin B12	155.4 ± 70.9	179.6 ± 71.9	206.6 ± 79.0	<0,001	A, B, C
Vitamin D	30.2 ± 9.0	39.9 ± 12.9	52.2 ± 18.7	<0,001	A, B, C

А: admission versus at 3 months, B: admission versus at 6 months, and C: at 3 months versus at 6 months.

**Table 3:** Correlations of laboratory variables with BI and mRS corrected for age and initial NIHSS.

		After 3 months	After 6 months
Homocysteine	BI	−.239	−.275
	mRS	.182	.231

B12	BI	.392^*∗∗*^	.375^*∗∗*^
	mRS	−.275	−342^*∗*^

Vitamin D	BI	.031	.193
	mRS	.049	−.231

*Note*. ^*∗*^*p* < 0.01. ^*∗∗*^*p* < 0.05.
